# Provision and Guidance for Postpartum Contraception - Ensuring Reproductive Rights during Times of Crises

**DOI:** 10.1055/s-0041-1729985

**Published:** 2021-05-12

**Authors:** Mariane Massaini Barbieri, Cassia Raquel Teatin Juliato, Fernanda Garanhani Surita

**Affiliations:** 1 Universidade Estadual de Campinas, Campinas, SP, Brazil

Reproductive choice and access to contraception are basic rights for all women, representing a major step forward in the improvement of gender equality. Sexual and reproductive health is paramount when considering public health, and has been directly affected by the COVID-19 pandemic, since these services suffered a reduction in their capacity for elective care, including consultations directed at contraceptive counseling. 1 This could lead to increase in the number of unplanned pregnancies, which has already been reported as above 50% of all pregnancies.

Postpartum contraception should be considered as an essential component of obstetric care. The immediate postpartum period, prior to hospital discharge is an excellent opportunity to counseling and providing contraceptives, particularly in those women who are unable to attend their follow-up appointments six weeks postpartum. 2 Approximately 40% of women do not attend their postpartum appointments, which favors an increased rate of unplanned and rapid repeated pregnancies (those which occur at intervals of fewer than 18 months). 3 4

Unplanned pregnancies strongly impact various aspects of women's lives, even more so when they occur during adolescence. A Brazilian study showed that women who fell pregnant between 16 and 19 years of age had lower schooling levels compared to those who did not fall pregnant [-2.8 years (95% CI: -3.2 to -2.3)], which was even more significant when pregnancy occurred between 11 and 15 years of age [-4.4 years (95% CI: - 5.5 to -3.3)]. These effects were more evident in women with three or more children. Income at 30 years of age was also 49% or 33% lower in women whose first child was born between 16-19 or 11-15 years old, respectively. 5

The new coronavirus pandemic has lead to emergency care services being prioritized in an attempt to reduce morbidity and mortality. Maternity hospitals have attempted to maintain humanized care during childbirth, which has highlighted their importance in approaching women and offering health education with an emphasis on reproductive health, specifically safe and effective postpartum contraception. 6 Ideally, counseling on available contraceptive methods should begin during antenatal care, via focus groups or during individual appointments. 7 Unfortunately, group activities have been restricted due to the need for social distancing, and the issue of postpartum contraception has not always been considered a priority during individual consultations. Therefore, the immediate postpartum period is an opportune and often unique moment to approach this important topic.

All available contraceptive methods that are considered appropriate for use during the postpartum period (etonogestrel (ENG) subdermal implants, intrauterine devices (IUDs), Depot Medroxyprogesterone Acetate and the progestogen-only pill) should be offered to women, with clear explanations of their advantages and disadvantages, duration of action, and the possibility of interruption or removal of the method according to patients' wishes. 8 All methods would ideally be presented through photos or posters, and in different orders so as to minimize coercion or promotion of one method over another. The counseling process must allow the woman free choice. 9

Long-acting reversible contraception (LARC) methods, such as ENG-implants and IUDs (which includes cooper IUD and levonorgestrel intrauterine releasing system) are considered the first choice for women. 10 LARCs are the most effective contraceptive methods, with a pregnancy rate of less than 1/1000 women/year, a higher degree of user satisfaction and higher continuation rates. Several international institutions, including the World Health Organization (WHO) and International Federation of Gynecology and Obstetrics (FIGO), recommend their introduction during the immediate postpartum period, as it presents a moment in which women are motivated to prevent new pregnancies, allowing hospital discharge with prevention methods in place. 11 It is important to highlight that this does not exempt the women from their postpartum follow-up appointments, which are mandatory and provide several other functions. 12

Post-placental intrauterine device insertion is an effective method for preventing unplanned and recurrent pregnancies, as well as to reduce healthcare visits, reducing costs and risks due to the pandemic. A study carried out in our service revealed that almost two thirds of women who opted for insertion of a post-placental IUD chose to maintain its use after one year. 13 Spontaneous expulsion of the IUD was the main reason for discontinued use. Another study showed that the insertion of post-placental IUDs prevented 88 pregnancies for every 100 women during a follow-up period of two years. The main disadvantage of post-placental intrauterine contraception is the higher expulsion rate, when compared to insertion after 40 days postpartum. However, a cost-benefit advantage is still considered. 14 Despite the practice of offering post-placental IUDs being routine in some settings, patient acceptance is still limited, most likely due to myths surrounding the use of IUDs, which are even more evident when considering the immediate postpartum period. It is important to highlight that some women present contraindications to IUD placement. Other considerations include, when prior counseling has not been offered, when time to allow the patient to consider the method is lacking, or should the healthcare team have limitations in offering any method at the opportune moment due to large numbers of simultaneous procedures or emergency situations. The insertion of a post-placental IUD is simple and safe, and requires minimum training, which is frequently offered by specialty college courses.

The ENG-implant is the most effective method, with a failure rate of 0.05%. It provides safe and long-term contraception in postpartum women, and does not interfere with breastfeeding or infant weight gain. 15 Studies relating to ENG-implant acceptance within the immediate postpartum period are few and far between in the scientific literature. A recent study performed during the peak of the first wave of coronavirus infection in Brazil, involving 151 women aged up to 24 years of age, showed high acceptance (76.2%). 16 The main disadvantage of the subdermal implant is its high cost; hence it is not routinely offered by healthcare services. During the immediate postpartum period, the principal advantage of the subdermal implant when compared to the IUD is the time scale in which a decision needs to be made regarding insertion, allowing the woman to make a more conscious decision. This is important when no prior antenatal counseling has been offered, as the woman has more time to consider and discuss her options, since the implant placement can be performed at any time before the hospital discharge. Insertion of the implant is simple and safe, however adequate training is required. 16 17

Assessment of risk/benefit, as well as other factors regarding the impact of hormonal contraception while breastfeeding must be considered. The WHO eligibility criteria show that the risk of using Depot Medroxyprogesterone Acetate in women less than 6 weeks postpartum outweighs the benefits (category 3). 18 Although there are some extenuating circumstances where its use during the immediate postpartum period has been reported, 19 it is more appropriate to advise and discharge women with a prescription for its use starting 6 weeks after delivery. Progesterone only pills may be started at any time after delivery. 12

The Lactational Amenorrhea Method (LAM) can be used in women who exclusively breastfeed on demand for up to six months postpartum, so long as they continue amenorrheic. Despite exclusive breastfeeding during this period, 20 to 56% of women restart menstruating earlier, decreasing its effectiveness. 20

During the first six weeks postpartum, barrier methods (male and female condoms) can be used without restriction. Their use should always be encouraged to prevent sexually transmitted diseases. 8

Difficulty in accessing health services and quality contraception in times of crisis negatively affects the reproductive health and life of women, especially vulnerable groups. The immediate postpartum period is important for implementation of reproductive healthcare, including counseling and the provision of contraceptive methods, such as the LARC, which has a long duration and is highly effective. 10 Offering other methods allows women a choice of the method as well the time scale to begin its use. This leads to better adherence, lower rates of unplanned and recurrent pregnancies, a more adequate interval between births and decreased maternal and child mortality. 9 Therefore, reproductive health counseling is a fundamental tool for health promotion. One should emphasize that maternal mortality has taken on a catastrophic dimension in Brazil during the coronavirus pandemic. 21

During a long pandemic such as in the current climate, which has required the restructuring of healthcare services to focus almost exclusively on coronavirus infection, the adequate advice on contraceptive methods for women in the postpartum period prior to hospital discharge is an essential tool to guarantee the health and rights of reproductive choice of each woman.
